# Perceptions of time constraints among primary care physicians in Germany

**DOI:** 10.1186/s12875-019-1033-5

**Published:** 2019-10-22

**Authors:** Olaf von dem Knesebeck, Sarah Koens, Gabriella Marx, Martin Scherer

**Affiliations:** 1 Institute of Medical Sociology, University Medical Center Hamburg-Eppendorf, Martinistr. 52, 20146 Hamburg, Germany; 2 Department of General Practice and Primary Care, University Medical Center Hamburg-Eppendorf, Martinistr. 52, 20146 Hamburg, Germany

**Keywords:** Primary care physicians, Germany, Time constraints, Time stress

## Abstract

**Background:**

Time constraints during patient visits play a major role for the work stress of primary care physicians. Several studies suggest that there is a critical situation in terms of time constraints in primary care in Germany. Therefore, the following research questions are addressed: (1) What is the time allocated and needed for different types of consultations among primary care physicians in an urban area in Germany? (2) What is the extent of time stress? (3) Are there differences in time stress according to physician characteristics (gender and length of experience) and practice type (single vs. group/shared practice)?

**Methods:**

Data stem from a face-to-face survey of primary care doctors in Hamburg and adjacent regions. A sample of 128 physicians stratified by a combination of physicians’ gender and length of experience (≤15 years or > 15 years) was used. Physicians were asked about the time needed (in minutes) to provide high quality of care for patients regarding six types of consultations: (1) new patient appointment, (2) routine consultation, (3) complete physical examination, (4) symptom-oriented examination, (5) check-up, and (6) home visit (without drive). Afterwards, they were asked about the average minutes allocated for the six consultations. Time stress was measured by calculating minutes needed minus minutes available.

**Results:**

Average perceived time needed was higher than time allocated for all six types of consultation. However, there were differences in the magnitude of time stress between the consultation types. Time stress was most pronounced and most prevalent in case of a new patient visit. No significant differences in time stress between male and female primary care physicians were found, while less experienced physicians reported more time stress than those with more experience (> 15 years). Physicians working in a single practice had less time stress than those working in a group or shared practice in case of a check-up visit.

**Conclusions:**

Perceived time needed is higher than time allocated for various types of consultation among primary care physicians in Germany. Time stress in primary care is particularly pronounced in case of new patient appointments. Early-career physicians are particularly affected by time stress.

## Background

The issue of adverse working conditions in primary care is of great importance as such conditions may have negative consequences for physicians and their patients [1–3]. Time constraints and pressure during patient visits play a major role for the work stress of primary care physicians. Moreover, time pressure adversely influences physicians’ diagnostic accuracy by increasing their stress response [4]. Several studies suggest that there is a critical situation in terms of time constraints in primary care in Germany [5–8].

In this regard, the international survey of the Commonwealth Fund showed that German primary care physicians were working about 51 h per week in 2009 of which 70% (i.e. 35.5 h) were spent with patient contacts [6]. Proportion of time spent with patients was higher in most of the other participating countries. Average reported time per patient contact in Germany was 7.8 min in 2006 [7] and increased to 9.1 in 2009 [6]. In another international study, consultation time was measured with a stop watch; average time for Germany was 7.6 min [9]. These values were lowest when compared to the other participating countries. Findings from the 2015 Commonwealth Fund survey furthermore showed that 45% of the German primary care physicians were dissatisfied with the time spent per patient [8]. They criticised that there was too much time required for administrative tasks and documentation [6, 10].

Such results are important as they give insight into working conditions of primary care physicians, but for a deeper understanding of time stress more differentiated measures and analyses are needed: First, to give consideration to the manifold patient contacts in primary care, different types of consultations (e.g. new patient visit, routine follow-up consultation, complete physical examination) should be addressed [5]. Second, it is useful to differentiate between time needed and time allocated and to calculate time stress by subtracting the first from the latter [11]. Third, it can be expected that there are differences according to physician characteristics like gender or length of experience and practice setting [5, 11–13].

Against this background, the following research questions will be addressed: (1) What is the time allocated and needed for different types of consultations among primary care physicians in an urban area in Germany? (2) What is the extent of time stress? (3) Are there differences in time stress according to physician characteristics (gender and length of experience) and practice type (single vs. group/shared practice)?

## Methods

### Study design and participants

Data stem from a face-to-face survey conducted between June 2018 and February 2019 as part of a study designed to examine clinical decision making. A sample of primary care doctors in Hamburg and adjacent regions of Lower Saxony and Schleswig Holstein was drawn from a comprehensive list of the Department of General Practice / Primary Care at the University Medical Center Hamburg-Eppendorf. Primary care physicians within a radius of two hours driving time from the University Medical Center Hamburg-Eppendorf were invited. To systematically examine differences between physician characteristics, the study population was stratified by a combination of physicians’ gender and length of experience as board certified general practitioner or internist (≤15 years or > 15 years) into four strata. For inclusion, the physicians had to be working at least 20 h per week with the main focus on primary care. Since information about length of clinical experience and working time was not available from the database, invited physicians were contacted per phone call to ascertain eligibility as well as willingness to participate and schedule an appointment for a 30 to 40 min in-person one-on-one interview in the physician’s practice during or directly after their normal practice hours. Participating physicians received a stipend to acknowledge their participation and to partly offset lost revenue. The final sample consisted of 64 female and 64 male primary care physicians; half of them with ≤15 years of clinical experience and half of them with > 15 years of clinical experience The participation rate of eligible doctors was 50.4%. Informed consent was signed by all study participants. According to a statement of the Ethics Committee of the Hamburg Medical Association (processing number: PV5663) no ethics approval was needed for the study.

### Measures

Following the procedure suggested by Linzer et al. [11] and Konrad et al. [5], physicians were asked about the time (in minutes) needed to provide high quality of care for patients regarding six types of consultations: (1) new patient appointment, (2) routine consultation, (3) complete physical examination, (4) symptom-oriented examination, (5) check-up, and (6) home visit (without drive). The first consultation type refers to a visit of a patient the doctor hasn’t seen before. The second refers to a routine follow-up visit of a patient with a pre-existing medical condition or a previously known consultation event. While in case of the third type, the whole body is examined, e.g. in case of a complex symptom constellation or a medical certificate, the fourth type refers to physical examinations to clarify circumscribed symptoms. A check-up aims at assessing health-related risks and screening health conditions. Home visits are for patients who are too ill or lack mobility to attend the practice. Afterwards, the physicians were asked about the average minutes allocated for the six types of consultations. As proposed by Konrad et al. [5] and Linzer et al. [11], time stress was measured by calculating minutes needed minus minutes available for each type of consultation.

### Analyses

Descriptive statistics (means (in minutes) and standard deviations) for time needed and allocated as well as for time stress are shown for the different types of consultation. Additionally, the percentage of physicians experiencing time stress was calculated. To examine the differences according to physician characteristics (gender and length of experience) and practice type (single vs. group/shared practice), t-tests were calculated. For these analyses, means, standard deviations, and significances (*p* values) are reported. Analyses were conducted with the statistical software IBM SPSS Statistics 25.0.

## Results

Mean age of the 128 participating primary care physicians was 54.5 years (females: 54.7 years, males: 54.3, physicians with more than 15 years of experience: 60.3 years, physicians with 15 or less years of experience: 48.6 years). Of the participants, 73% were general practitioners, 18% internists, and 9% were general practitioners and internists. 35% of the physicians worked in a single practice, the remaining 65% in a group or shared practice.

Table 1 shows the perceived time allocated, time needed, and time stress among the primary care physicians. Means of time allocated vary between 5.7 min for a symptom-oriented examination and 19.7 min for a check-up. For all consultation types, time needed is higher than time available. This holds especially true for a new patient visit (difference between time needed and time allocated 4.4 min), whereas time stress is least pronounced in case of a symptom-oriented examination (0.7 min) and a home visit (0.8 min). Large standard deviation (6.2) indicates a large variability of differences between time needed and time available for home visits among the physicians (range: − 35 min to 20 min). More than 50% of the physicians reported time stress in case of a new patient visit. This rate is lowest for a home visit (16.4%).

**Table 1:** Perceived time allocated, time needed, and time stress (time needed minus time allocated) among primary care physicians in Germany (*N* = 128)

	Time allocated (in minutes, means, SD)	Time needed (in minutes, means, SD)	Time stress (in minutes, means, SD)	Physicians with time stress (%)
New patient appointment	16.2 (6.9)	20.6 (7.1)	4.4 (6.1)	53.1
Routine consultation	8.9 (3.9)	10.1 (3.8)	1.2 (3.2)	39.8
Complete physical examination	11.1 (5.3)	12.7 (5.7)	1.6 (4.3)	36.7
Symptom-oriented examination	5.7 (3.2)	6.4 (3.4)	0.7 (2.8)	27.3
Check-up	19.7 (8.9)	22.6 (8.6)	2.9 (6.1)	39.8
Home visit (without drive)	18.0 (8.6)	18.8 (7.8)	0.8 (6.2)	16.4

*SD* Standard deviation

Table 2 shows that there are no significant differences (*p* < 0.05) in time stress between male and female primary care physicians. In terms of clinical experience, there are two significant differences: Physicians with ≤ 15 years of experience have more time stress in case of a routine consultation and a complete physical examination than those with more than 15 years of experience. Although not reaching statistical significance, less experienced physicians also report more time stress in the other types of consultations. Physicians working in a single practice tend to have less time stress than those working in a group or shared practice. These differences are significant in case of a check-up visit.

**Table 2:** Time stress among primary care physicians according to gender, experience, and practice type: in minutes (means, standard deviations) and significances (p)

	Males (n = 64)	Females (n = 64)	p*	Experience ≤ 15 years (*n* = 64)	Experience > 15 years, n = 64)	p*	Single practice (*n* = 45)	Group/shared practice (*n* = 83)	p*
New patient appointment	4.8 (6.1)	3.9 (6.0)	0.39	5.3 (6.4)	3.3 (5.5)	0.06	3.5 (6.4)	4.8 (5.8)	0.26
Routine consultation	1.1 (3.3)	1.2 (3.1)	0.90	1.8 (3.2)	0.6 (3.1)	0.03	0.9 (3.4)	1.3 (3.1)	0.54
Complete physical examination	1.5 (4.0)	1.6 (4.7)	0.84	2.5 (4.5)	0.6 (4.1)	0.01	0.9 (3.0)	1.9 (4.9)	0.23
Symptom-oriented examination	1.0 (2.3)	0.3 (3.3)	0.20	0.7 (3.3)	0.6 (2.3)	0.79	0.6 (2.5)	0.7 (3.0)	0.89
Check-up	3.9 (7.5)	1.9 (4.1)	0.06	3.9 (6.6)	2.0 (5.4)	0.08	1.5 (6.1)	3.7 (6.0)	0.04
Home visit (without drive)	1.1 (6.2)	0.6 (6.3)	0.67	1.8 (7.2)	−0.2 (4.9)	0.06	0.7 (6.1)	0.9 (6.3)	0.83

*t-test

## Discussion

Primary care physicians “… are expected to form partnerships with patients and their families, address complex acute and chronic biomedical and psychosocial problems, provide preventive care, coordinate care with specialists, and insure informed decision making that respect patients’ needs and preferences.” [14] Time constraints impede this challenging task.

Based on a stratified sample of 128 primary care physicians, this study showed that average perceived time needed was higher than time allocated for all six types of consultation under study (new patient appointment, routine consultation, complete physical examination, symptom-oriented examination, check-up, and home visit). However, there were differences in the magnitude of time stress (i.e. more time is needed than available) between the consultation types. Time stress was most pronounced and most prevalent in case of a new patient visit. Perceived time needed, allocated and time stress showed considerable variance between physicians.

A previous international comparative study analysing time needed and allocated for three different types of visits (new patient, routine visit, and complete physical exam) revealed that German primary care physicians had the least time allocated and needed for each type of appointment compared to physicians in the UK and in the U.S. [5]. Consistently, German primary care physicians asked less questions and gave less advice than British or American doctors [15, 16]. Previous studies from the U.S., UK, and Germany also showed that time stress in primary care is particularly pronounced in case of new patient appointments [5, 11]. Especially with new patients, sufficient time is needed for the different dimensions of the medical history (recent history, personal history, psychosocial history, allergological anamnesis and family history) and for a profound view of the objective global physical state.

In terms of physician characteristics, no significant differences in perceived time stress between male and female primary care physicians were found, while less experienced physicians reported more time stress than those with more experience (> 15 years). Additional analyses (not shown in detail) revealed that the differences in time stress according to experience were mainly due to lower estimations of the minutes allocated among the less experienced physicians whereas reports on time needed were quite similar between the two groups. Differences between male and female physicians were found in questioning, counselling, and visit length in previous studies from different countries [12, 13]. However, the few studies investigating gender differences in time stress in primary care, brought inconsistent results [5]. Our analyses also do not support the assumption that female doctors may have more time stress as they conduct longer visits [12].

Primary care physicians working in a single practice had less time stress than those working in a group or shared practice in case of a check-up visit. Although, differences according to practice type were not significant for the remaining five consultations, time stress was consistently lower in solo practices. This finding is in line with a study comparing different primary care practice settings in the U.S. [11].

There are a couple of methodological limitations that need to be considered when evaluating our results. Analyses were based on a relatively small regional sample of primary care physicians working in an urban area in Germany (Hamburg and adjacent regions of Lower Saxony and Schleswig Holstein). Although Steinhauser et al. [17] found only few differences in working conditions of German primary care physicians between rural and urban areas, our findings cannot be generalized to other regions. As it was intended to systemically examine differences between physician characteristics, a stratified sampling procedure was chosen, further limiting generalizability. Moreover, almost half of the eligible physicians refused to participate or were not available. Thus, a selection bias cannot be ruled out, although a response rate of more than 50% is quite good for a physician survey [18]. Such a possible bias can be expected to lead to an underestimation of time stress because physicians with more time pressure are less likely to participate in studies [19]. It is important to keep in mind that all information on time was based on self-reports and we were not measuring consultation length [20]. Although one can argue that time stress is a subjective phenomenon and self-reports are adequate, our definition of time stress in a way is crude. Assessing time stress by calculating minutes needed minus minutes available was originally suggested by Linzer et al. [11] and Konrad et al. [5]. By using this procedure, it is possible to calculate the percent of physicians experiencing time stress and the magnitude of time stress in minutes (Table 1). In future studies, magnitude of time stress among primary care physicians should be investigated using larger, nationwide samples. Furthermore, consequences of time stress for quality of care should be examined using prospective designs.

## Conclusions

Perceived time needed is higher than time allocated for various types of consultation among primary care physicians in Germany. Time stress in primary care is particularly pronounced in case of new patient appointments. Early-career physicians are particularly affected by time stress. Adverse working conditions like time stress can have negative consequences for physicians and their patients [21–23]. We assume that the perceived time pressure actually reflects current health care issues in Germany. Increasing time pressure and workload are associated with features of the remuneration system for primary care. In this regard, it has been proposed that remuneration should be decoupled from the provision of individual services and personal uptake, e.g. by introducing payment of a capitation fee per registered patient [24]. This could be one measure to reduce the high contact rate among primary care physicians in Germany as well as time pressure.

## Data Availability

The dataset used and analyzed is available from the corresponding author on reasonable request.
